# Parenting Styles and Attention Deficit Hyperactivity Disorder in the South Asian Population: A Narrative Review

**DOI:** 10.7759/cureus.74967

**Published:** 2024-12-02

**Authors:** Panna Patel, Jaspreet Behl, Tahia Karim, Sainamitha R Palnati, Saajan Bhakta

**Affiliations:** 1 Department of Psychiatry, Yuma Regional Medical Center, Yuma, USA; 2 Department of Psychiatry, University of Texas Southwestern Medical Center, Dallas, USA; 3 Department of Research, Kansas College of Osteopathic Medicine, Wichita, USA

**Keywords:** adhd, asian, attention deficit hyperactivity, ethnicity, mental health, mental illness, parent-child interaction therapy, parenting styles, south asian

## Abstract

The relationship between cultural practices and mental health is particularly complex in diverse communities. Attention deficit hyperactivity disorder (ADHD), a prevalent neurodevelopmental condition, remains underdiagnosed and untreated in South Asian populations, often due to persistent stigmas and systemic barriers. While global awareness of ADHD is increasing, research examining its connection with parenting styles in South Asian families is limited. Cultural norms, societal expectations, and family dynamics unique to this group may influence how ADHD symptoms are perceived, managed, and addressed. This narrative review seeks to fill this gap by (i) identifying existing studies on ADHD and parenting styles in South Asians; (ii) exploring cultural and familial factors that may affect the expression of ADHD symptoms; and (iii) highlighting areas for future research to enhance understanding of ADHD in the South Asian community. By synthesizing these findings, this review aims to identify research gaps and inform future interventions and clinical practices tailored to the needs of South Asian families.

## Introduction and background

Attention deficit hyperactivity disorder (ADHD) is a neurodevelopmental disorder characterized by persistent patterns of inattention, impulsivity, and hyperactivity that interfere with daily functioning. It is categorized into three subtypes: inattentive, hyperactive-impulsive, and combined presentations. The inattentive type involves difficulty focusing, organizing tasks, following through on instructions, and frequent forgetfulness, which can lead to academic and social challenges. The hyperactivity/impulsive type is marked by restlessness, excessive talking, difficulty sitting still, and impulsive behaviors like interrupting or acting without considering consequences. The combined type includes symptoms from both categories and is the most common subtype of ADHD. These symptoms can significantly disrupt a child’s academic, social, and emotional development, often persisting into adulthood if left untreated [1,2].

Although ADHD has been extensively researched in many populations, much of the existing literature focuses on Western contexts, leaving gaps in understanding how ADHD manifests and is managed in diverse cultural groups [3]. South Asians, a growing demographic in the USA, represent a culturally rich population that includes individuals from countries such as Afghanistan, Bangladesh, Bhutan, India, Maldives, Nepal, Pakistan, and Sri Lanka. Despite their increasing presence, research addressing mental health issues, particularly ADHD, within this community is scarce [3]. The stigma surrounding mental illness persists in South Asian communities, with cultural attitudes frequently viewing mental health problems as signs of personal weakness or familial failure [4-7]. These stigmatizing beliefs deter help-seeking behaviors and exacerbate disparities in mental health care access and utilization [4-6,8,9]. Furthermore, systemic barriers, such as language difficulties, lack of culturally competent services, and limited awareness of available mental health resources, compound these challenges [4-6,8,9]. Together, these factors contribute to the underdiagnosis and undertreatment of ADHD and other mental health conditions among South Asians, perpetuating disparities in care and outcomes.

Parenting styles, influenced by cultural values and societal norms, may play a crucial role in shaping the development and management of ADHD symptoms [10-12]. Within South Asian families, collectivist values, respect for authority, and an emphasis on academic excellence strongly influence parenting practices [13,14]. There are four main parenting styles: authoritarian, authoritative, permissive, and uninvolved. Authoritarian parents enforce strict rules without explanation, focusing on obedience and discipline, which can lead to well-behaved, often anxious or aggressive children who struggle with decision-making. Authoritative parents, in contrast, maintain a nurturing relationship while setting clear expectations, explaining the reasons behind their rules, and encouraging open communication. This approach fosters confidence, responsibility, and emotional well-being in children. Permissive parents are warm and indulgent, with few rules or expectations, leading to children who are often impulsive and struggle with self-regulation, although they typically have good social skills. Uninvolved parents are emotionally detached and provide minimal guidance or support, which can result in children who are self-sufficient but may struggle with emotional and academic challenges [15]. Additionally, extended family members, like grandparents, often play a significant role in caregiving, contributing to a collective approach to parenting that blends traditional and modern influences [16].

Within South Asian families, parenting practices are often influenced by a duality: balancing emotional support with high expectations for academic and personal success. This reflects cultural priorities emphasizing both collectivist values and family honor, which place significant pressure on parents to ensure their children excel academically [14]. The patriarchal structures common in South Asian communities further shape parenting dynamics, with gender disparities influencing expectations and treatment of children [5]. For instance, boys may face pressures to succeed academically and professionally, while girls may experience distinct expectations tied to traditional family roles [5]. Additionally, extended family members, like grandparents, often play a significant role in caregiving, contributing to a collective approach to parenting that blends traditional and modern influences [16].

Despite the growing understanding of ADHD, research examining how South Asian cultural, societal, and familial dynamics influence the diagnosis and management of ADHD remains scarce. Misinterpretation of ADHD symptoms within the context of cultural norms often delays diagnosis and leads to ineffective management strategies [3,4]. Moreover, the limited representation of South Asians in mental health research leaves critical gaps in developing culturally tailored interventions and support systems.

This review seeks to address these gaps by exploring the relationship between ADHD symptoms and parenting styles within South Asian families. It examines how cultural stigma, societal expectations, and family dynamics influence ADHD’s presentation, diagnosis, and management. By synthesizing existing research, this study aims to inform future interventions and clinical practices tailored to the unique needs of South Asian families, ultimately contributing to improved mental health outcomes for this underrepresented population.

## Review

Methods

This systematic review was conducted using electronic databases such as PubMed and Google Scholar between April 15, 2024, and May 15, 2024. The search focused on peer-reviewed studies published in English between 2000 and 2024 to encompass advancements in ADHD diagnostic criteria (e.g., the transition from the Diagnostic and Statistical Manual of Mental Disorders (DSM)-IV to DSM-5), improvements in research methodologies enabled by technological developments, and evolving societal attitudes toward mental health. Technological progress during this period, such as the introduction of neuroimaging, electronic health records, and advanced statistical tools, improved ADHD diagnosis and understanding. Societal shifts included increasing stigmatization of mental health conditions and a growing focus on minority populations like South Asians, making this time frame highly relevant to the review’s objectives.

An advanced search strategy used Boolean operators (“AND” and “OR”) and included keywords such as “South Asian”, “Asian”, “South Asian children”, and “ethnicity”. The search was restricted to peer-reviewed studies in English within the specified time frame, resulting in 133 articles. To refine the search, additional terms such as “Mental illness” OR “mental health” AND “ADHD” OR “Attention Deficit Hyperactivity Disorder” were added, narrowing the pool to 39 articles. To further tailor the results to the review’s objectives, terms like “Parenting styles” OR “PCIT (Parent-Child Interaction Therapy)” were introduced, but this specific search yielded no results. Additional combinations, such as “ADHD and South Asian”, “Parenting styles and South Asians”, and “Mental Health and parenting styles in Asians”, were iteratively explored to ensure a comprehensive search. Ultimately, 13 studies were selected after applying eligibility criteria.

The inclusion criteria required studies to focus on South Asian populations, address ADHD or its symptoms in relation to parenting styles, and be peer-reviewed articles published in English between 2000 and 2024. Eligible studies needed to directly involve South Asian children, adolescents, or families, exploring ADHD within the context of parenting. Exclusion criteria included non-peer-reviewed articles, opinion pieces, and letters to the editor, as well as studies unrelated to South Asians, ADHD, or parenting styles. Articles with non-standardized diagnostic measures or insufficient methodological rigor were also excluded. Duplicates were removed to ensure a robust dataset.

The search and selection process began with screening titles and abstracts of the 133 retrieved articles, conducted independently by two reviewers. Articles that met the inclusion criteria were reviewed in full text. To refine the selection further, the combination of “Mental health” and “Parenting styles” reduced the pool to 39 articles. Additional review was conducted for depth of analysis and specificity to the research objectives, and the final selection was narrowed to 13 studies. These included a range of study designs: one systematic review and meta-analysis, one retrospective cohort study, nine cross-sectional studies, one non-randomized pre-post intervention study, and one case-control study.

**Figure 1:**
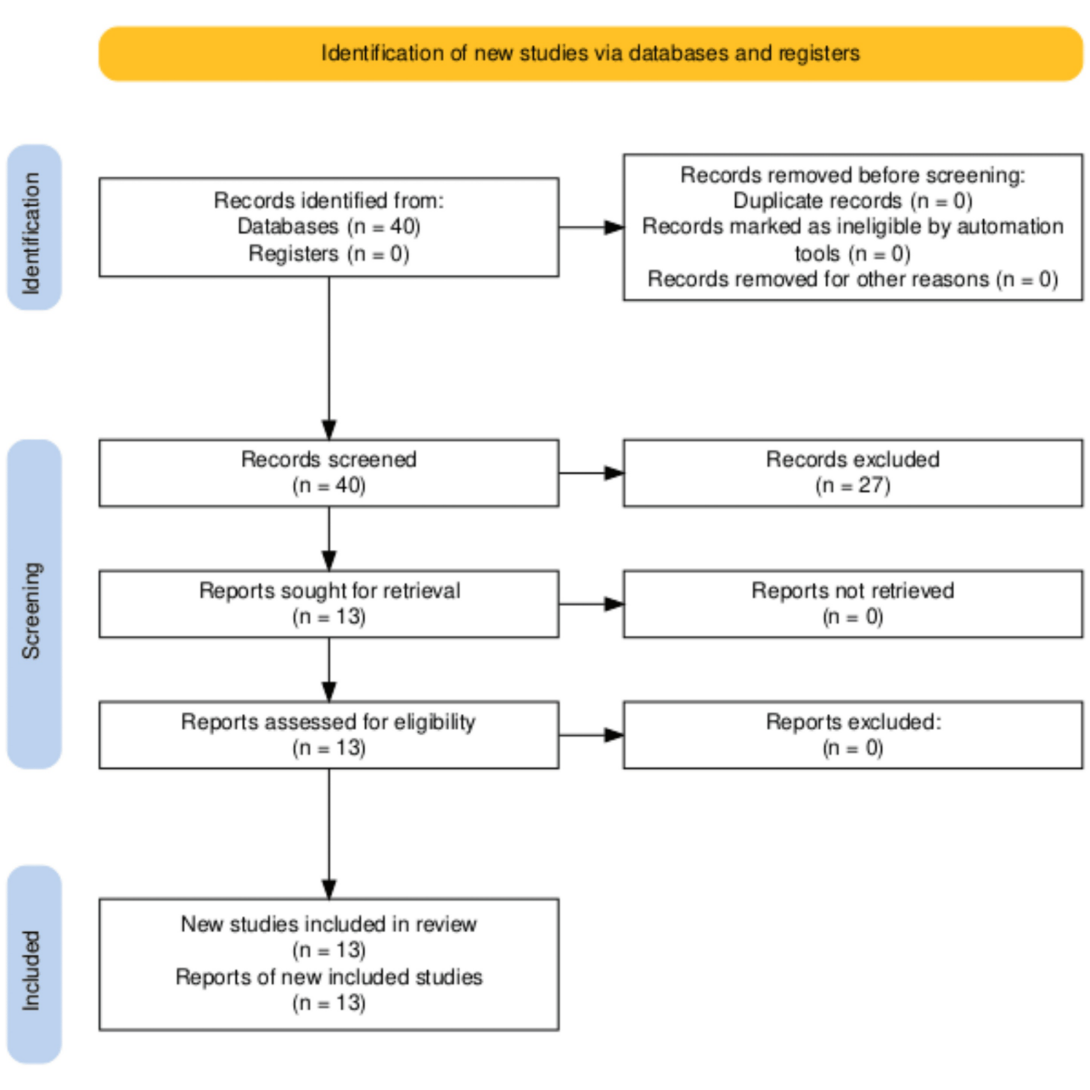
PRISMA flow diagram of the summarized search strategy PRISMA: Preferred Reporting Items for Systematic Reviews and Meta-Analyses

The Preferred Reporting Items for Systematic Reviews and Meta-Analyses (PRISMA) guidelines were followed to ensure methodological transparency and consistency while minimizing selection bias. The PRISMA flow diagram (Figure 1) outlines the search and screening process in detail. The iterative approach to searching and refining keywords was necessitated by the limited body of research specifically addressing ADHD and parenting styles within South Asian populations. This methodology enabled the identification of relevant studies, contributing to a better understanding of the intersection between ADHD, mental health, and parenting in South Asian families.

Literature review

This narrative review aims to deepen the understanding of ADHD within the South Asian community by examining the interplay between cultural factors and parenting styles. While previous sections highlighted the challenges of diagnosis and treatment in this population, the following discussion will delve into specific aspects: the prevalence of ADHD, the impact of stigma surrounding mental illness, and how distinctive parenting styles may shape the manifestation of ADHD symptoms. By exploring these aspects, the aim is to provide insights into the unique experiences of South Asians with ADHD and to identify culturally relevant strategies for support and intervention.

Parenting Styles and ADHD

Ellis and Nigg aimed to look at the relationship between parenting practices and ADHD, emphasizing the unique contributions of maternal and paternal behaviors to the manifestations of ADHD symptoms in children. The methodology of the study assessed children (n = 181) aged 6-12 years old for ADHD via a semistructured clinical interview completed by a parent, and researchers used the DSM-IV criteria to place them in groups. The study classified the children into four groups: non-ADHD control group (n = 52), ADHD inattentive type (n = 24), ADHD combined type (n = 71), and ADHD - not otherwise specified/borderline (n = 34). Parents were also assessed for ADHD using self-reported structured interviews, as well as assessed for parenting practices by completing the Alabama Parenting Questionnaire [17].

Maternal inconsistent discipline was significantly associated with ADHD combined type (ADHD-C), with a partial η2 = 0.14 (p < 0.001). This finding persisted even after controlling for oppositional defiant disorder, conduct disorder, and parental ADHD symptoms, suggesting that inconsistent maternal discipline uniquely contributes to ADHD-C symptoms. Paternal low involvement, on the other hand, was associated with ADHD symptoms across subtypes, with a partial η2 = 0.092 (p = 0.002). However, when paternal ADHD symptoms were controlled, the association diminished to marginal significance (p = 0.051; partial η2 = 0.051). These findings underscore the differential impacts of maternal and paternal parenting practices on ADHD symptomatology [17].

Interestingly, Ellis and Nigg observed that the relationship between paternal low involvement and ADHD symptoms was more pronounced in the inattentive/disorganized domain than in hyperactivity or other behavioral domains. Paternal inconsistent discipline also showed a unique association with child inattention symptoms, remaining significant regardless of covariates (p < 0.05). These results suggest that paternal parenting behaviors may play a critical role in shaping the inattention symptoms of ADHD, highlighting the importance of involving fathers in interventions targeting this domain [17].

The study’s use of the Alabama Parenting Questionnaire allowed for the assessment of multiple parenting domains, revealing that maternal inconsistent discipline was related to all behavioral outcomes, including inattention (r = 0.26, p < 0.001), hyperactivity (r = 0.31, p < 0.001), oppositionality (r = 0.32, p < 0.001), and conduct problems (r = 0.27, p < 0.001). Similarly, paternal involvement was inversely related to inattention (r = -0.27, p < 0.01), suggesting that increased paternal engagement might mitigate these symptoms. This dimension-specific analysis highlights the nuanced relationship between parenting practices and ADHD symptomatology [17].

Despite the robust statistical analyses, several limitations warrant consideration. First, the reliance on self-reported parenting practices and ADHD symptoms introduces potential biases, including underreporting or social desirability effects. For example, fathers’ responses may have understated the extent of low involvement or inconsistent discipline, as data from paternal reports were missing in a substantial portion of the sample. Missing data were addressed using the expectation-maximization algorithm, but the reliance on available data without full imputation may have underestimated paternal effects. Second, the cross-sectional design precludes causal inference. While the findings suggest that inconsistent discipline and low involvement are associated with ADHD symptoms, the directionality of this relationship remains unclear. Longitudinal studies are needed to disentangle whether parenting practices exacerbate ADHD symptoms or whether challenging behaviors in children elicit inconsistent or disengaged parenting [17].

Another limitation is the homogeneity of the sample, which predominantly included White participants. Parenting practices and their effects on child development are deeply influenced by cultural and societal norms. For example, South Asian families often exhibit parenting styles characterized by high expectations, collectivist values, and hierarchical family structures [12,13]. These cultural differences may mediate or moderate the relationship between parenting practices and ADHD symptoms, limiting the generalizability of the findings to diverse populations. Future research should aim to replicate these findings in culturally diverse samples, particularly among underrepresented populations such as South Asians, to explore how cultural norms shape parenting behaviors and their impact on ADHD [17].

The inclusion of parental ADHD symptoms as a covariate adds strength to the findings, demonstrating that the associations between parenting practices and child ADHD were not fully explained by shared genetic predispositions. However, the reliance on self-reported ADHD symptoms in parents may have led to an underestimation of these effects. Comprehensive assessments of parental ADHD, including collateral reports or clinical evaluations, are necessary to further clarify these dynamics [17].

Similarly, Moghaddam et al. conducted a case-control study to examine parenting styles in parents of children with ADHD compared to those without ADHD, providing insights into how different parenting approaches may interact with ADHD symptoms in Zahedan, Iran. Using Baumrind’s Parenting Styles Questionnaire, the study assesses 75 parents of children with ADHD and 75 parents of children without ADHD, finding notable differences in parenting styles between the two groups [18].

The results revealed that parents of children with ADHD had significantly lower permissive parenting scores (M = 27.4, SD = 4.4) compared to the control group (M = 29.0, SD = 4.2), with a significant difference (p = 0.019). This suggests that parents of children with ADHD exert greater control over their children’s behavior, possibly as a response to the challenges posed by ADHD symptoms. Additionally, the authoritarian parenting score was significantly higher among parents of children with ADHD (M = 23.5, SD = 6.1) than in the control group (M = 20.3, SD = 3.9), with p < 0.001. This finding highlights the demanding yet less responsive approach often adopted by these parents, which may inadvertently exacerbate ADHD symptoms by failing to provide the emotional support and flexibility needed by children with ADHD. Interestingly, the authoritative parenting scores did not differ significantly between the two groups (p = 0.646), indicating that the balance of responsiveness and demandingness associated with authoritative parenting may not be disrupted in families with ADHD [18].

Moghaddam et al. noted that the more controlling (authoritarian) parenting style in the ADHD group could worsen symptoms due to its rigid and punitive nature, which is less adaptive to the needs of children with ADHD. This aligns with broader research suggesting that high parental demands coupled with low responsiveness can impair the development of self-regulation and exacerbate behavioral difficulties. Conversely, the lower permissive scores in the ADHD group reflect an effort by parents to impose structure and control, possibly in reaction to their children’s hyperactive and impulsive behaviors. However, this may lead to a counterproductive dynamic, where increased control amplifies oppositional or inattentive behaviors [18].

Despite the valuable insights, the study had limitations that must be considered. The reliance on self-reported questionnaires for assessing parenting styles introduces potential biases, such as social desirability bias, where parents might underreport punitive or authoritarian behaviors. Moreover, the small sample size (n = 150) limits the generalizability of the findings, particularly across diverse cultural contexts. The study was conducted in Zahedan, Iran, with a culturally homogenous sample, making it unclear whether the findings apply to other populations, including South Asian families in different sociocultural settings. Parenting styles are deeply influenced by cultural norms and values, and South Asian families, for example, may exhibit distinct practices shaped by collectivist values and hierarchical family structures [12,13]. Another limitation is the cross-sectional design, which precludes causal inferences. It remains uncertain whether authoritarian parenting contributes to the development or worsening of ADHD symptoms or whether children with ADHD elicit more controlling behaviors from their parents. Future studies employing longitudinal designs could better clarify the directionality of these relationships [18].

Jones et al. sought to examine whether the parenting styles that college students experienced mediated the relationship between the students’ ADHD symptoms and academic adjustment to college. The sample included 200 undergraduate students, aged 18 to 42 years old, from a large public university in the midwestern USA. The students were primarily Caucasian (79%), but the sample did include other ethnicities including African American (15%), Asian/Pacific Islander (3%), and other (5%). Students’ ADHD symptoms were measured by Conners’ Adult ADHD Rating Scale-Self-Report (CAARS-S:S). Parenting styles were measured by students completing a Parental Authority Questionnaire about their primary caregiver that measured three different parenting styles: permissive, authoritarian, and authoritative. Academic adjustment of the students was measured by a self-reported questionnaire titled “The Student Adaptation to College Questionnaire” (p. 254) [19].

Jones et al. found that academic adjustment and ADHD symptoms were strongly correlated, r = -0.44, p < 0.001. Looking at parenting styles, Jones et al. noted that academic adjustment declined in students with higher symptoms of ADHD in both low levels of authoritarian parenting (t(116) = -5.19, p < 0.001) and authoritative parenting (t(196) = -3.67, p < 0.01). Higher levels of both authoritarian, t(116) = -2.11, p < 0.001, and authoritative parenting, t(116) = -5.50, p < 0.001, also showed a similar pattern of a decrease in academic adjustment when ADHD symptoms increased. It could be possible that other psychological conditions that are associated with higher ADHD symptoms in adults may invalidate parenting styles [19]. Given the samples’ ethnicities, the researchers did not consider other ethnic diversities, so their findings cannot be generalized to different culture-specific parenting styles and ADHD. The study also based the parenting styles solely on the student’s perspective and did not reach out to the parents to talk about their own behavior, which could lead to bias.

Parenting Styles Within the South Asian Community

Focusing on South Asian parenting styles, Yim sought to examine the relationship between Asian cultural values, parenting styles, and children’s perceived competence in Hong Kong. The study also aims to understand the cultural and parenting style differences between local Chinese and South Asian parents living in Hong Kong. Additionally, the study sought to understand cultural and parenting style differences between local Chinese and South Asian parents. Participants included 48 local Chinese parents, 49 South Asian parents, and a total of 105 children (24 local Chinese and 81 South Asian) aged five to six years old. Within the South Asian group, the sample was diverse, comprising Indonesians (33%), Nepalese (11%), Pakistanis (34%), and Filipinos (22%), collectively referred to as “South Asian” due to the limited sample size [12].

The study utilized three self-reported questionnaires: two questionnaires assessed the parents’ adherence to Asian cultural values and parenting practices, and one Pictorial Scale of Perceived Competence and Acceptance (PSPCSA) for children to measure their perceived competence in cognitive, physical, peer, and maternal acceptance. Components of Asian cultural values included collectivism, achievement, and humility. Statistical analyses, including multiple linear regression models, revealed significant findings: the authoritative parenting style correlated positively with adherence to Asian cultural values in the South Asian sample (R^2^ = 0.597). In contrast, there was no significant relationship between adherence to cultural values and authoritarian (R^2^ = 0.145) or permissive (R^2^ = 0.268) parenting styles [12].

Yim suggested that the prominence of authoritative parenting among South Asian parents in Hong Kong might reflect a degree of cultural accommodation to the host society. This adaptive behavior aligns with acculturation theory, which posits that immigrant groups often adopt values or behaviors of the host culture to navigate and integrate into the new environment. Components like humility (β = 0.490) within Asian cultural values were particularly influential in predicting the adoption of authoritative parenting, highlighting how cultural ideals shape parenting approaches [12].

However, the study has notable limitations. The small sample size of South Asians restricts generalizability, as the representation of different South Asian ethnic groups was uneven, with certain subgroups (e.g., Nepalese) being underrepresented. The use of self-reporting questionnaires introduced potential biases, such as social desirability, where participants might overreport behaviors perceived as socially acceptable. Additionally, the reliance on a collective term (“South Asian”) to describe a highly heterogeneous population obscures important intergroup differences [12]. Future studies should aim to expand the sample size and disaggregate findings by specific South Asian ethnicities to provide a more nuanced understanding of the cultural and contextual factors shaping parenting styles.

Moreover, Yim’s study was conducted in Hong Kong, where cultural, socioeconomic, and systemic factors likely differ significantly from those in the USA. For instance, Hong Kong’s cultural landscape is heavily influenced by Chinese collectivist traditions, which may shape both parenting behaviors and the acculturation experiences of South Asian families differently than in the USA. Therefore, Yim’s findings may not fully generalize to the experiences of South Asians in the USA, where the cultural and systemic contexts differ substantially. Yim’s study also did not address the potential influence of socioeconomic status, language proficiency, or length of residence in Hong Kong, all of which could affect the acculturation process and parenting behaviors. Given that the South Asian participants were largely settled in lower socioeconomic districts, the findings may reflect environmental or financial constraints influencing parenting choices rather than purely cultural factors [12].

Future studies should address these limitations by including larger, more diverse samples and exploring South Asian parenting styles within different host countries, particularly the USA, to examine how varying cultural and systemic contexts shape parenting practices and child outcomes. Expanding the scope of research in this manner could help disentangle the influence of cultural values from external environmental factors, providing a more comprehensive understanding of South Asian parenting behaviors across different settings.

Similarly, Ho et al. investigated cultural differences in parenting styles and their impact on child behavior. The study utilized data from the National Longitudinal Survey of Children and Youth (NLSCY), which included 14,990 children aged four to 11 years old among various Canadian ethnic groups, including South Asians, collected between 1998 and 1999. The methodology involved extensive interviews in English or French, covering aspects of child behavior, parental practices, and family variables. Multilevel analyses were conducted to examine parental harshness and its association with child aggression and emotional problems, as reported by parents and teachers [20].

The study revealed intriguing insights into parenting practices within South Asian Canadian families. Ho et al. found that South Asian Canadian families demonstrated a statistically significant decrease in parental harshness compared to their European Canadian counterparts (South Asians: α = 0.61 and European Canadians: α = 0.76; β = -0.147, p < 0.05). This finding suggests that South Asian Canadian parents reported employing less harsh parenting practices than their European Canadian counterparts. Additionally, parental harshness was found to have differential effects on child aggression based on cultural background. For European Canadian children, higher levels of parental harshness were associated with increased aggression at home but decreased aggression in school (X^2^(1) = 132.3, p < 0.001). In contrast, South Asian Canadian children showered lower levels of aggression in school even at higher levels of parental harshness, indicating a contrasting relationship between parenting style and children's behavior across cultural groups (X^2^(1) = 0.46, ns) [20].

Moreover, in examining parenting reports of child aggression, significant cultural differences emerged. East Asian, South Asian, and Caribbean Canadian children were associated with lower levels of parent-reported aggression compared to European Canadian children (East Asian: β = -0.014, South Asian: β = -0.014, Caribbean: β = -0.027, all p < 0.05). This suggests that cultural background influences both parental perceptions of child behavior and the prevalence of aggressive behaviors reported within each group. Furthermore, the interaction between parental harshness and cultural groups indicated varying slopes in their relationship between harsh parenting and child aggression across East Asian, South Asian, and Caribbean Canadians compared to European Canadians. European Canadians were seen to have the steepest slope (X^2^(1) = 198.4, p < 0.001), with South Asian children not being significantly different from European Canadian children [20].

Despite the comprehensive approach, the reliance on parental self-reports for assessing parenting styles and child behavior rather than actual observation introduced potential biases, such as social desirability or cultural norms influencing reporting tendencies. Further limitations inherent in this study include the focus on Canadian demographics and the use of data from the late 1990s, which may not fully reflect contemporary dynamics in parenting practices or cultural adaptations. These limitations underscore the importance of critically evaluating cultural influences on parenting styles, especially in contexts where cultural values and societal expectations may shape perceptions and responses to child behavior and mental health issues like ADHD [20].

Ho et al. underscore the complexity of cultural influences on parenting practices and child behavior within diverse Canadian ethnic groups. The findings highlight that cultural background not only shapes parenting styles, such as the level of harshness, but also moderates the impact of parenting on child outcomes like aggression. These insights are crucial for understanding how cultural norms and parenting behaviors interact to influence child development and mental health outcomes, including potential implications for conditions like ADHD within specific cultural contexts [20].

Stewart et al. aimed to explore the association between perceived parental styles and practices with academic achievement, psychosocial functioning, and relationship harmony among adolescents in Bangladesh. This study included 130 males and 82 females (n = 212) between the ages of 14 and 15 from grade school, focusing exclusively on Muslim adolescents born in Bangladesh. Methodologically, participants completed translated Bangla versions of various scales measuring parenting styles (warmth and dominating control), academic achievement, psychosocial functioning (self-esteem, life satisfaction, relationship harmony), and self-derogatory ideation [21].

Key findings indicated significant gender differences in perceptions of parental styles and their effects. Females reported higher levels of parental dominating control (F(1, 203) = 6.34, p = 0.01) and supervision (F(1, 203) = 21.94, p =.001) compared to males. Perceived parental warmth was positively correlated with supervision for females, whereas for males, it was more strongly associated with dominating control (z = 1.98, p = 0.03). Females who perceived higher dominating control exhibited lower academic achievement, while males showed the opposite trend (z = 2.76, p = 0.004). Conversely, females with higher perceptions of supervision reported better academic achievement, whereas no such association was found for males (z = 2.21, p = 0.02) [21].

Additionally, males who perceived parental warmth reported greater relationship harmony outside the family context, whereas perceived dominating control was negatively linked to relationship harmony for males (z = 1.72, p = 0.05). Self-derogatory ideation mediated the relationship between perceived parenting styles and academic achievement specifically for females, explaining 22% of the variance (F(5, 75) = 4.14, p = 0.002). This mediation model suggested that perceived dominating control adversely affected academic outcomes through increased self-derogatory thoughts among females [21].

Limitations of this study include reliance on self-reported data, which can introduce response biases, and the exclusive focus on Muslim adolescents from Bangladesh, limiting generalizability to broader cultural contexts. The study of gender imbalance, with more males than females, might have influenced the comparative analysis between the sexes. Furthermore, being an older study, societal changes and evolving parental practices could affect the applicability of these findings to contemporary contexts. This study contributes to the understanding of how perceived parenting styles impact adolescents’ development differently across genders within the specific cultural context of Bangladesh. The findings underscore the importance of parental warmth and supervision in promoting academic success and psychological well-being among adolescents, highlighting the detrimental effects of dominating parental behaviors, particularly for females [21].

Saleem et al. investigated the interplay between mother and father parenting styles and their impact on distress tolerance and psychological distress among Pakistani university students. The study utilized a stratified sampling strategy to recruit participants between the ages of 16 and 25, focusing on self-reported measures including the Distress Tolerance Scale and the Depression Anxiety Stress Scale (DASS-SF). Pearson correlations were used to examine associations, and regression analyses explored the predictive power of parenting styles on distress tolerance and psychological distress, considering interaction effects [13].

Consistent findings from Saleem et al. revealed that overprotectiveness among parents was significantly linked to poorer psychological functioning in Pakistani young adults. Specifically, both overprotection and rejection were associated with lower distress tolerance and higher psychological distress; emotional warmth from parents, however, showed no significant associations with either distress tolerance or psychological distress. Regression analysis indicated significant effects. For distress tolerance problems, the interaction between emotional warmth and rejection significantly predicted outcomes (β = 0.42, p < 0.001), where higher emotional warmth attenuated the negative impact of parental rejection. Similarly, for psychological distress, this interaction effect was also significant (β = 0.45, p < 0.001), suggesting that higher emotional warmth buffered against the adverse effects of parental rejection on psychological distress [13].

Despite these insights, the study has several limitations. It relied solely on self-reported data, which may introduce inaccuracies in reporting parenting styles and psychological outcomes. The narrow age range of the sample limits generalizability to older age groups, and the urban-centric sample composition may not represent rural or diverse urban populations in Pakistan or other South Asian countries. Additionally, the measures of distress tolerance and psychological distress were highly overlapping, precluding detailed mediation analysis to explore the mechanisms underlying the observed association [13].

Prevalence of ADHD Within the South Asian Community

Adams et al. evaluated the racial-ethnic differences in ADHD diagnosis and treatment from adolescence to early adulthood of Asians, Hispanics, African Americans, and Caucasians. The study analyzed a cohort of 4,216,757 youths, with 5.18% identified as Asian (n = 218,564) who had commercial insurance coverage. The authors had defined ADHD diagnosis as having at least two medical visit claims with an ICD code for ADHD, spaced at least two days apart, or one or more medical claims with an ICD code for ADHD accompanied by at least one prescription claim for ADHD medication. Researchers calculated prevalence ratios (PRs) by comparing ADHD diagnosis and treatment prevalence in each racial-ethnic group using Caucasian youth as a reference, as researchers had found Caucasian youth have the highest rates of ADHD diagnosis and treatment [4].

The study reported that Asians had a low prevalence rate of 3.1% for ADHD diagnosis between ages 12 and 14 (PR = 0.29, 95% CI (0.28, 0.30)), indicating that Asian youth were 71% less likely to be diagnosed with ADHD compared to Caucasian youth, who had a prevalence rate of 10.6%. The study further reported that Asians had a significantly lower prevalence rate of ADHD treatment once diagnosed (80.8%, PR = 0.94) compared to Caucasian youths (86.1%) [4].

However, as stated before, the study was not specific to the South Asian population, and its broad sample may not be directly applicable to South Asian subgroups. The research also lacked consideration of cultural factors that are particularly relevant to this group, such as cultural mistrust and experiences of interpersonal racism. Additionally, Adams et al. focused only on commercially insured youths, using diagnostic codes from insurance claims, which do not capture detailed information on symptoms or diagnostic methods. As a result, the sample may not accurately represent the broader US population in terms of socioeconomic status or racial/ethnic diversity. There is also a potential underestimation of ADHD diagnosis and treatment rates among populations that lack commercial insurance, receive care in community-based settings or schools, or pay out of pocket for services [4].

While Adams et al. looked at ADHD diagnosis and treatment among a large cohort, Dissanayake et al. examined differences in obsessive-compulsive disorder (OCD), ADHD, and anxiety self-/parent-reported diagnosis rates within a smaller and more culturally specific sample. The methodology consisted of recruiting East Asian, South Asian, and White participants between the ages of six and 17 years old while visiting the Ontario Science Centre between 2008 and 2009. The participants’ race and ethnicity were measured based on the self-reported or parent-reported race and ethnicity of their maternal and paternal grandparents [8].

Out of the total participants (n = 8,927), the South Asian population had the least number of participants (n = 730), followed by East Asian youth (n = 1301) and most occurring being Caucasian youth (n = 6896). South Asians were found to have 88% lower odds of self-reporting their ADHD diagnosis when compared to Caucasian youth. Dissanayake et al. suggested that the lower prevalence of ADHD within the South Asian community could be due to barriers that prevent access to mental health care. The study utilized self-/parent-reported diagnosis instead of clinician-reported information, which was a significant limitation. The study also recruited from a science museum that required an entrance fee, which limited the pool of participants and therefore limited each population’s representative sample [8].

Looking at ADHD prevalence within South Asians specifically, Ranjan et al. completed a systematic review and meta-analysis including 61 studies that were conducted between 1980 and 2023 within South Asian countries to estimate the prevalence of ADHD within South Asian countries [22].

Ranjan et al. included 58,231 children and adolescents, and out of this pool, 3,950 children showed symptoms of ADHD, with an estimated prevalence rate of 61.9 out of 1,000 population (95% CI (47.8, 77.6), p < 0.001). Although statistically significant (p < 0.001), the estimated prevalence rate was low in comparison to other countries, including Africa and Spain, with studies reporting the prevalence of ADHD in the child and adolescent population to be higher at 7.47% and 6.8%, respectively [22].

Also, Ranjan et al. examined various sub-groups, including gender and urban versus rural populations, for the prevalence of ADHD. The study found that there was a higher prevalence rate for ADHD diagnosis in males (82.6 per 1,000, 95% CI (62.3, 105.3)) than in females (48.3 per 1,000; 95% CI (31.6, 68.0)) within South Asian countries, similar to what was found within the USA. Ranjan et al. discussed that the lower prevalence rate of females can be due to their presentation of symptoms: the study identified that males tend to show their ADHD symptoms more externally, like running around and jumping, while females show their symptoms more internally, like inattentiveness. This presentation could be the reason for a later or even underdiagnosis of ADHD in females when compared to males. The study also reported that there was a higher prevalence rate of ADHD symptoms in children and adolescents living in urban settings (72.2 per 1,000; 95% CI: 55.0-91.6 per 1,000) compared to rural (42.9 per 1,000; 95% CI: 24.2-66.4 per 1,000) in South Asian countries (p = 0.0509). The study discussed that children living in rural settings have more barriers, including a lack of mental health services and professionals that are available to them, increased stressors, and decreased early intervention services and special education when compared to children in urban settings. The study also reported that there is decreased recognition of ADHD symptoms in rural settings, which may lead to an underdiagnosis due to a lack of education [22].

However, given that the study was a meta-analysis of the primary studies, there was a lack of sampling frames, which limited generalizability to the general population and served as a significant limitation. The study also obtained data for the prevalence rates through responses from interviews by parents, teachers, or self-reports, which may contribute to an underestimation bias. It is also imperative to note that there were several barriers reported by the study in relation to the low prevalence of ADHD within the South Asian community. These barriers included access to mental health care, socioeconomic status, self- and social stigma of mental illness, limited access to treatment, and urban versus rural settings. Barriers, including lack of education to recognize symptoms as well as lack of mental health services that are available, are seen specifically in the prevalence rates of gender and urban versus rural settings. These barriers could contribute to the underestimation bias and potentially impact the prevalence rates that were estimated by the research team [22].

While there is a lack of research to contextualize if South Asian parents of children with ADHD implement a specific parenting style, steps have been taken to incorporate South Asian culture within parenting interventions for children with ADHD. Shah et al. sought to describe and develop a culturally contextualized parent skills training intervention (PSTI) for Indian families. The methodology included using 41 families that were divided into five groups, with each group receiving a weekly group PSTI for 10 weeks. Of the 41 families, 36 families attended more than three sessions. Out of the 36 children that participated in the study, 18 children received medication for ADHD as well as PSTI, whereas the other 18 children received only PSTI. The Vanderbilt ADHD Diagnostic Parent Rating Scale (VADPRS) was used to assess ADHD symptoms [23].

Implementation of PSTIs overall was shown to yield an improvement in inattention (p < 0.001), hyperactivity (p = 0.007), and conduct problems (p < 0.002) between the pre- and post-intervention VADPRS scores. Academic performance (p < 0.001) and classroom behavior (p = 0.001) were also shown to yield a statistically significant reduction in the number of problem areas in the post-intervention VADPRS scores. The study also noted no change in the VADPRS score between the PSTI-only group and the PSTI-plus medication group (t = -2.819, p = 0.008). Additionally, the researchers analyzed the perceived helpfulness and difficulties of the culturally contextualized PSTIs. A total of 97.2% of the Indian families (n = 35 families) who had participated in the PSTI reported an increase in awareness of ADHD, and 86% (n = 31 families) reported a decrease in stress. However, 50% of the families (n = 18 families) found it difficult to sustain the PSTI strategies long-term, and 33.3% (n = 12 families) reported difficulties in getting other family members or spouses to implement the PSTI strategies. Shah et al. suggested that these challenges the families faced could stem from a lack of awareness or stigma surrounding ADHD, as well as the structured nature of the intervention, which some families and older children involved in the study might have found to be burdensome [23].

Despite key findings, several limitations should be noted from this study, including a small sample size (n = 36 families) that can limit the generalizability of the key findings to a broader population. The study utilized parent-reported measures for assessing the efficacy of PSTI, which can introduce potential biases in the reporting. The study also lacked a medication-only control group that could have been used to compare the efficacy of the PSTI [23]. Without the control group, it is difficult to determine whether the PSTIs’ observed effects were due to the intervention itself or the combined effects of the PSTI and the medication that children took for their ADHD. Future research should investigate the clinical effects of PSTIs within South Asian communities by including a control group, which would provide stronger evidence of the efficacy of the culturally contextualized PSTI.

Stigma of ADHD/Mental Illnesses Within the South Asian Community

A significant challenge that was identified within the South Asian community was the negative stigma around mental illness. Arora et al. conducted a cross-sectional survey to examine perceived and personal stigma on attitudes toward professional help across the South Asian community. The methodology included the Perceptions of Stigmatization by Others for Seeking Help Scale (PSOSH), a 5-point Likert type scale, as well as the Social Distance Scale (SDS), a seven-item self-reported questionnaire, to a group of South Asian college students (n = 160), aged 18-22 at a university in the southern USA. Attitudes toward professional help were assessed by the Attitudes Toward Seeking Professional Psychological Help Scale (ATSPPH) [7].

Arora et al. found that South Asian college students in the USA held increased negative attitudes associated with seeking professional help for mental illness (p < 0.001), which included a tendency to avoid association with individuals with mental health issues. The study also found that the male gender was associated with more negative attitudes toward seeking psychological help (p < 0.05) due to the traditional male role that is expected from South Asian men. However, no significant association was found between seeking professional help and the perceived stigma by others (p = 0.156). Arora et al. suggested that the way the participants answered the questions on perceived stigma was focused more on anticipated reactions from others toward the participants’ mental illnesses than how the participants react toward others with mental illnesses [7].

It is important to acknowledge that this study had a small sample size (n = 160) of college students, which cannot be used to generalize across the entire South Asian population at large. The study also did not take into account other cultural factors that could influence attitudes toward professional help seeking, which included acculturation, generational status, family dynamics, or prior mental health experiences. More importantly, the study looked at attitudes toward professional help but did not directly look at the help-seeking behavior of the participants. Future studies should seek to measure actual engagement with mental health services in a larger sample of South Asian participants for a more impactful and meaningful understanding of South Asians’ experiences with mental health struggles [7].

Likewise, Cadet et al. sought to understand if Indian mothers’ socioeconomic status along with their understanding and attitudes toward ADHD correlated to their child’s diagnosis of ADHD in India. The study used a cross-sectional survey design. Surveys were completed by 100 Indian mothers with children aged four to 12 years old between January and August 2017. The researchers had the mothers check off the CDC ADHD checklist to assess whether their child had any symptoms, as well as obtain information regarding the mother’s knowledge about ADHD and what instances they would seek help for their child’s behaviors [5].

Out of the 100 mothers, 19% had less than a high school degree, 53% had a high school degree, and 28% had more than a high school education. A total of 35% of the mothers who participated had knowledge regarding ADHD, and 42% had knowledge of how to handle their child’s behavior. Researchers identified several factors that were significantly associated with six or more behaviors participants self-reported in the CDC ADHD checklist, including the gender of the child (p = 0.014), the state in which the mother was raised (p = 0.035), the mother’s knowledge on handling the child’s behavior (p = 0.008), the mother's knowledge on ADHD (p = 0.000), and the mother’s willingness to seek professional help (p = 0.032). The behaviors that were most common were hyperactivity (75%), becoming distracted (74%), fidgeting (70%), and inattentiveness (69%). The study also noted that there was no significance in ADHD diagnosis and the parent responsible for the child’s behavior (p = 0.705) or addressing the child’s behavior (p = 0.690). This finding may suggest that broader cultural factors, beyond parental responsibility, could influence the child’s ADHD diagnosis [5].

Cadet et al. reported that in Indian culture, the mother is the primary caregiver of the child. With such responsibility given in society, if the child were to have a mental illness like ADHD, it was a direct reflection of the mother’s caregiving and parenting style. This perception of the mother’s caretaking leads to the potential increase in negative stigma around mental illness within the community. However, the self-reporting nature of the study may have led to inaccuracies as the mothers could have recalled their child’s behavior differently, leading to information bias [5]. Additional limitations include the potential for cultural bias, as the participants may have responded in a manner that avoided the negative stigma associated with mental illnesses, as well as the small sample size (n = 100), which limits the generalizability of the study.

Limitations 

It is important to acknowledge the limitations that were encountered while doing this literature review. A major limitation was the lack of studies done on ADHD within the South Asian population, particularly in relation to the parenting styles of South Asians who have children with ADHD. To address this limitation, our search period was expanded to include publications within the last 24 years to encompass more studies. Addressing the lack of studies is important for advancing the understanding of ADHD and its impact on the South Asian community. Another notable limitation of this literature review was the selection criteria for the included studies. The review only considered published, peer-reviewed articles written in English, which excluded potentially relevant studies that were written in different languages or published without peer review. This restriction could have narrowed our range of findings as well as limited the inclusion of more diverse perspectives. The lack of research highlights the importance of further examining the relationship between specific parenting practices, such as authoritative, authoritarian, and permissive styles, and ADHD symptoms in South Asian individuals above the age of 18, which could significantly improve our understanding of how ADHD impacts this community.

## Conclusions

Research on ADHD within the South Asian population, particularly concerning parenting styles, remains limited. This review highlighted the critical need to address the underdiagnosis and undertreatment of ADHD in this demographic, where cultural stigmas, systemic barriers, and limited awareness significantly hinder mental health care access. South Asian families often face challenges rooted in cultural norms, such as the prioritization of academic success, collectivist values, and hierarchical family structures, all of which influence how ADHD symptoms are perceived and managed. These factors, compounded by a lack of culturally competent mental health services, contribute to the persistent disparities in ADHD diagnosis and treatment. A key finding from this review was the predominance of authoritarian parenting styles within South Asian families. While this approach may provide structure, it can exacerbate ADHD symptoms by placing excessive pressure on children or dismissing their struggles as behavioral issues. However, the limited research specific to South Asians makes it challenging to draw definitive conclusions about the role of parenting styles in managing ADHD. Future studies should focus on exploring culturally specific parenting practices and their impact on ADHD outcomes to better understand this complex dynamic.

To improve support for South Asian families, healthcare providers and policymakers must address the barriers identified in this review. Culturally tailored interventions, such as parenting skills training programs that consider South Asian cultural values, could help bridge the gap in ADHD management. Efforts to reduce stigma through community outreach and education are equally essential. Moreover, increasing the representation of South Asians in mental health research is crucial for developing interventions that reflect the unique needs of this population. Addressing the cultural and systemic barriers to ADHD diagnosis and treatment within South Asian communities is critical for fostering better outcomes. By expanding research, promoting culturally competent care, and prioritizing stigma reduction, future efforts can improve the lives of South Asian families navigating the challenges of ADHD. This review underscored the importance of integrating cultural understanding into mental health practices to create more equitable and effective solutions for underserved populations.
